# Adjuvant chemotherapy in average-risk adult medulloblastoma patients improves survival: a long term study

**DOI:** 10.1186/s12885-020-07237-x

**Published:** 2020-08-12

**Authors:** E. Franceschi, S. Minichillo, A. Mura, A. Tosoni, M. Mascarin, C. Tomasello, S. Bartolini, A. A. Brandes

**Affiliations:** 1 Department of Medical Oncology, Azienda USL, Bologna, Italy; 2 SOSD Oncologia Integrata del Giovane e Radioterapia Pediatrica, Centro di Riferimento Oncologico (CRO) IRCCS, Aviano, Italy; 3 Department of Medical Oncology, Papardo Hospital, Messina, Italy

**Keywords:** Medulloblastoma, Chemotherapy, Survival, Average-risk

## Abstract

**Background:**

Medulloblastoma is extremely rare in adults. The role of chemotherapy for average-risk adult patients remains controversial. Surgery and radiotherapy provide a significant disease control and a good prognosis, but about 25% of average-risk patients have a relapse and die because of disease progression. No data in average-risk adult patients are available to compareradiotherapy alone and radiotherapyfollowed byadjuvant chemotherapy.

**Methods:**

We analyzed 48 average-risk patients according to Chang classification diagnosed from 1988 to 2016.

**Results:**

Median age was 29 years (range 16–61). Based on histological subtypes, 15 patients (31.3%) had classic, 15 patients (31.3%) had desmoplastic, 5 patients (10.4%) had extensive nodularity and 2 patients (4.2%) had large cells/anaplastic medulloblastoma. Twenty-four patients (50%) received adjuvant radiotherapy alone and 24 (50%) received radiotherapy and chemotherapy. After a median follow-up of 12.5 years, we found that chemotherapyincreases progression-free survival (PFS-15 82.3 ± 8.0% in patients treated with radiotherapy and chemotherapyvs. 38.5% ± 13.0% in patients treated with radiotherapy alone *p* = 0.05) and overall survival (OS-15 89.3% ± 7.2% vs. 52.0% ± 13.1%, *p* = 0.02). Among patients receiving chemotherapy, the reported grade ≥ 3 adverse events were: 9 cases of neutropenia (6 cases of G3 neutropenia [25%] and 3 cases of G4 neutropenia [13%]), 1 case of G3 thrombocytopenia (4%) and 2 cases of G3 nausea (8%).

**Conclusions:**

Our study with a long follow up period suggests that adding adjuvant chemotherapy to radiotherapy might improve PFS and OS in average-risk adult medulloblastoma patients.

## Background

Medulloblastoma is rare in adults (less than 1% of primitive CNS tumors) with an incidence of 0.6–1 case per million per year [1–3].

Correct staging is an important prognostic factor by influencing therapeutic program. Fundamental staging examinations are brain/spinal MRI before and after (48 h) surgery and CSF cytology performed 15–20 days after surgery. Tumors are classified for their extension and site of origin (T) and absence or presence of metastasis inside or outside the neuraxis (M) according to Chang’s staging system [4, 5]. Correctly staged, patients are usually divided into average and high risk groups.

The average-risk group presents no metastasis (M0) and no residual disease after surgery (residual disease has been defined > 1.5 cm 2). High-risk patients have metastases and/or residual disease and often unfavorable histology (large cells/anaplastic) [3].

For the treatment of pediatric average-risk patients, Packer et al. proposed a schedule which is now considered the standard treatment of pediatric average-risk population [6, 7].

The role of chemotherapy for average-risk adult patients remains controversial. In literature, there are no data if adding chemotherapy to radiotherapy improves the results.

Therefore, the possibility to associate chemotherapy to the standard treatment is still an open question and currently adjuvant chemotherapy could be evaluated in patients with poor risk histology (large cells/anaplastic).

Thus, we performed a retrospective analysis about outcomes of consecutive average-risk adult patients followed in our Institution and treated with radiotherapy alone or with radiotherapy plus chemotherapy.

## Methods

Patients included in our data warehouse were ≥ 16 years of age, had histologically confirmed medulloblastoma and underwent adjuvant radiotherapy with or without chemotherapy. Average-risk was defined as postsurgical residual ≤1.5 cm^2^ and no metastatic disease (M0) according to Chang’s classification.

The patients were staged with brain MRI and, whenever possible, also spine MRI before surgery. In all patients postsurgical MRI with contrast enhancement was routinely used to define residual disease within 48–72 h from surgery. Spine MRI was performed after surgery if not available before. CSF cytology was obtained at least 15 days far from surgery. Radiotherapy was administered with the dose of 36 Gray (Gy) in 20 fractions on the cranio-spinal axis plus a boost of 18 Gy in 10 fractions on the posterior cranial fossa (total dose 54 Gy). Chemotherapy regimens were: cisplatin (25 mg/ m^2^ on days 1–4) plus etoposide (40 mg/ m^2^ on days 1–4) or carboplatin (300 mg/m^2^ on day 1) plus etoposide (60 mg/ m^2^ on days 1–3).

### Statistical analysis

Data are reported as medians, ranges and frequencies. T-Test, Fisher’s exact test and Pearson’s chi-squared test were used. Survival data were computed through Kaplan-Meier procedure and were analyzed by means of the log-rank test. PFS and OS were computed from the time of surgery to the first progression or death, respectively, or to the date of the last follow-up or contact. Patients lost to follow-up were censored in the survival analysis. The SPSS (Version 13.0 for Windows; SPSS Inc., Chicago, IL, USA) was used as statistical package. Two-tailed *P* values less than 0.05 were considered significant.

## Results

We included 48 average-risk patients diagnosed from 1988 to 2016. Median age was 29 years (range 16–61), M/F ratio was 26 (54.2%)/22 (45.8%). The most represented histologies were: classic in 15 patients (31.3%), desmoplastic in 15 patients (31.3%), extensive nodularity in 5 patients (10.4%) and large cells/anaplastic in 2 patients(4.2%).

The patients were homogeneously distributed on two groups: 24 (50%) received only adjuvant radiotherapy and 24 (50%) also received chemotherapy. No differences were found among the two groups for age (*P* = 0.361), gender (*P* = 1.000) and histology (*P* = 0.702).

Patients’ characteristics are summarized in Table 1.

**Table 1:** Patients’ characteristics

	Chemotherapy	No Chemotherapy	Total
N	24	24	48
Mean Age	29 (range: 16-61)	31 (range: 16-57)	30 (range: 16-61)
M/F	13/11	13/11	26/22
Histology			
- Classic	7 (29.2%)	8 (33.3%)	15 (31.3%)
- Desmoplastic	6 (25.0%)	9 (37.5%)	15 (31.3%)
- Extensive Nodularity	3 (12.5%)	2 (8.3%)	5 (10.4%)
- LCA	1 (4.2%)	1 (4.2%)	2 (4.2%)
- Unknown	7 (29.2%)	4 (16.7%)	11 (22.9%)

### Safety

Data on toxicities are available for all patients. Toxicities were classified according to CTCAE v4.0. Among patients receiving chemotherapy, the reported grade ≥ 3 adverse events were: 9 cases of neutropenia and, particularly, 6 cases of G3 neutropenia (25%) and 3 case of G4 neutropenia (13%), 1 case of G3 thrombocytopenia (4%) and 2 cases of G3 nausea (8%) for a total of 12 grade ≥ 3 adverse events. Grade ≥ 3 toxicities related to radiotherapy alone were: 1 case of G3 hearing loss(4%), 2 cases of G3 neutropenia (8%) and 2 cases of G3 thrombocytopenia (8%) for a total of 5 grade ≥ 3 adverse events. No differences were found in the total number of grade ≥ 3 adverse events among the two groups (*P* = 0.069). Endocrinopathy (mild increase in TSH and prolactin) was found in only a patient treated with RT alone. No secondary malignancies were reported.

### Survival

After a median follow-up of 151.5 months (95% CI 124.5–178.5), 14 patients had disease progression and 10 patients died, 9 due to disease progression and one for other causes (considered as censored at the time of the event). Relapse sites were spinal, bone, cerebellum and brain.

### Progression-free survival

Median PFS was 9 years in patients who received radiotherapy and was not reached in those who received radiotherapy and chemotherapy. We found that adding chemotherapy increased PFS (HR 0.334; 95% CI 0.105–1.068, *p* = 0.05). This benefit was greater after 10 years from diagnosis: the rate of patients without progression at 10 and 15 years (PFS-10 and 15) was 82.3% ± 8.0% in the radiotherapy and chemotherapygroup versus 38.5% ± 13.0% in the radiotherapy group (Table 2).

**Table 2:** PFS and OS rates at 5, 10, 15, 20 years between patients treated with RT + CT and RT alone

	RT+CT	RT
**PFS-5**	86.9% ± 7.1%	87.3% ± 6.9%
**PFS-10**	82.3% ± 8.0%	46.2% ± 13.1%
**PFS-15**	82.3% ± 8.0%	38.5% ± 13.0%
**PFS-20**	82.3% ± 8.0%	38.5% ± 13.0%
**OS-5**	95.2% ± 4.6%	95.7% ± 4.3%
**OS-10**	89.3% ± 7.2%	74.1% ± 10.3%
**OS-15**	89.3% ± 7.2%	52.0% ± 13.1%
**OS-20**	89.3% ± 7.2%	41.6% ± 14.0%

### Overall survival

**Fig. 1:**
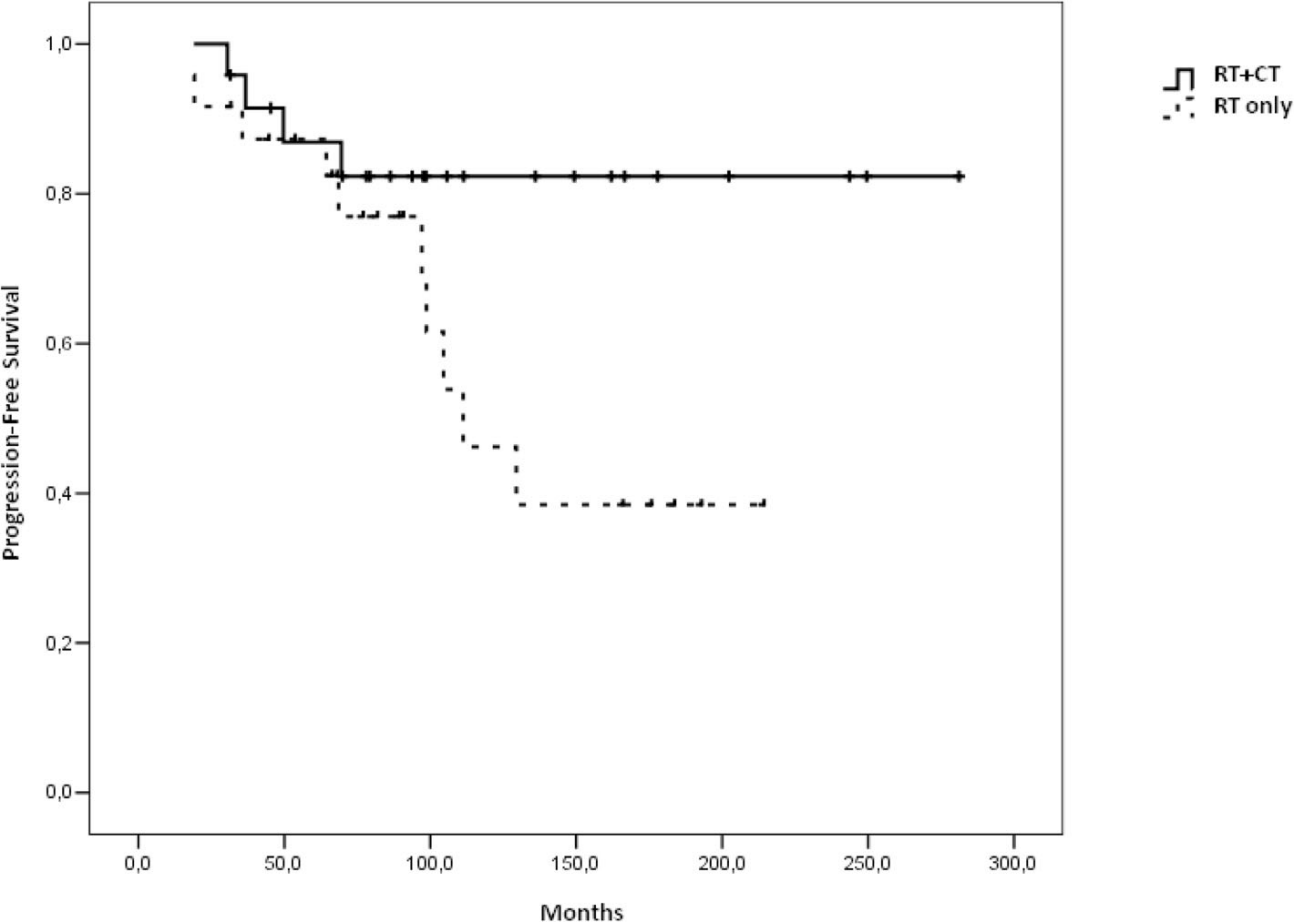
PFS according to treatment

**Fig. 2:**
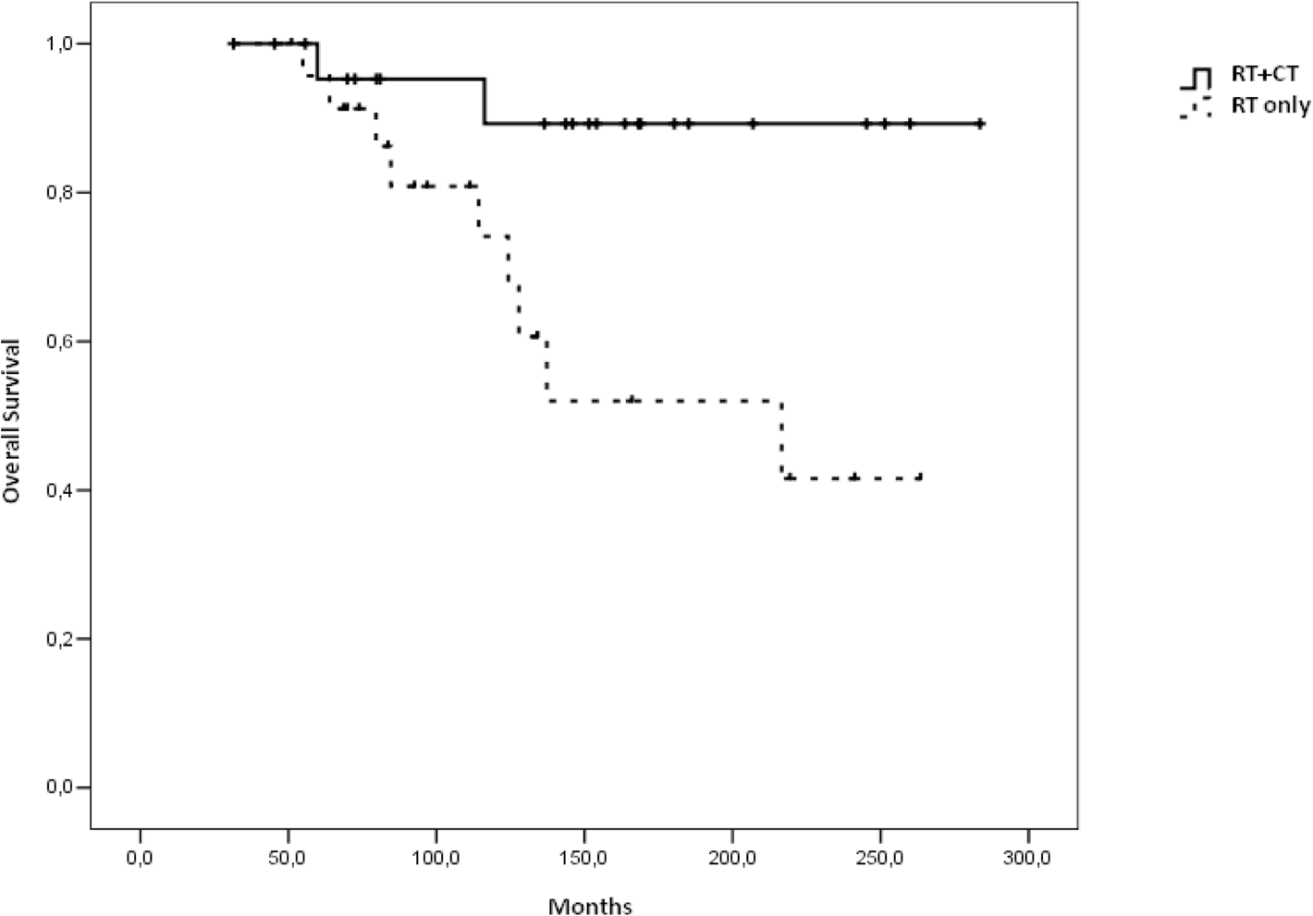
OS according to treatment

Median OS was 18 years (95% CI 89.0–344.1) in patients who received radiotherapy alone and was not reached in patients treated with radiotherapy and chemotherapywith a significant survival benefit in adding chemotherapy (HR 0.187; 95% CI 0.040–0.872, *p* = 0.02). This benefit was considerable with a longer follow up: the percentage of patients alive at 10 and 15 years (OS-10 and OS-15) were 89.3% ± 7.2% (radiotherapy and chemotherapygroup) vs. 74.1% ± 10.3% (radiotherapy group) and 89.3% ± 7.2% (radiotherapy and chemotherapygroup) vs 52.0% ± 13.1% (radiotherapy group) respectively (Table 2). Survival curves are reported in Figs. 1 and 2.

## Discussion

In average-risk patients the standard treatment includes radical surgery and radiation therapy. In Table 3 are summarized all related studies. In the management of young average-risk medulloblastoma patients, the possibility of adding chemotherapy has been regarded as an attempt to reduce total dose of radiotherapy delivered to brain and spinal cord and to limit toxic effects and long-term sequelae such as growth, neuro-cognitive and endocrinologic impairment. Packer et al. reported positive results in their trial in which children with non-disseminated medulloblastoma were treated with postoperative reduced-dose craniospinal irradiation (23.4 Gy in 13 fractions) with a boost to the posterior fossa (31.8 Gy in 17 fractions) with concomitant vincristine and adjuvant chemotherapy with lomustine, vincristine and cisplatin. They reported PFS rates at 3 and 5 years of 86 and 79% respectively, which are comparable with those obtained with full-dose radiotherapy alone. This schedule resulted in better tolerance and good safety and it currently represents the standard treatment of average-risk patients older than 3 years and younger than 18 years [6, 7].

**Table 3:** Main studies about the treatment of average risk medulloblastoma patients

Study	Therapy	Results
Packer et al	reduced-dose craniospinal radiation therapy (23.4 Gy) and 55.8 Gy of local radiation therapy plus concomitant vincristine chemotherapy and adjuvant lomustine, vincristine, and cisplatin chemotherapy	PFS 86% ± 4% at 3 years and 79% ± 7% at 5 years
Padovani et al	radiotherapy vs radio + chemotherapy	standard-risk disease could be treated with radiochemotherapy, reducing doses of RT
Greenberg et al	radiotherapy + POG protocol/Packer protocol	adults on POG protocol seemed to have less nonhematologic toxicity; on the Packer protocol appeared to have shorter median survival and greater toxicity than did children
Friedrich et al	radiotherapy + chemotherapy with lomustine, vincristine and cisplatin	EFS4 and OS4 were 68% ± 7% and 89% ± 5%. Peripheral neuropathy (74%) and haematotoxicity (55%) during maintenance chemotherapy appear to be more common in adults than in children
Beier et al (NOA-07)	craniospinal irradiation with vincristine, followed by 8 cycles of cisplatin, lomustine, and vincristine	radio-polychemotherapy did lead to considerable toxicity and a high amount of dose reductions
Kortmann et al	ARM 1: neoadjuvant chemotherapy with ifosfamide, etoposide, intravenous high-dose methotrexate, cisplatin, and cytarabine before radiotherapy ARM 2: immediate postoperative radiotherapy, with concomitant vincristine followed by 8 cycles of maintenance chemotherapy consisting of cisplatin, CCNU, and vincristine	maintenance chemotherapy would seem to be more effective in low-risk medulloblastoma Neoadjuvant chemotherapy was accompanied by increased myelotoxicity of the subsequent radiotherapy

In average risk adult population, the role of chemotherapy is still matter of debate.

Due to the rarity of the disease in adults, data in literature are few and derive mostly from retrospective and small series studies [8]. Randomized trials are not available.

Moreover, a long follow up period is needed to evaluate both PFS and OS.

Thus, data from retrospective studies including patients with homogeneous treatments and a long follow up period are essential to provide data.

A large retrospective analysis by Padovani et al. found no survival difference between average-risk patients treated with radiotherapy alone (axial doses ≥34 Gy) and patients treated with radiotherapy in combination to chemotherapy (axial doses <34Gy). This study was limited by heterogeneous chemotherapeutic regimens and data collected from different centers [9].

The role of chemotherapy is controversial due to high toxicity and the absence of randomized trials in average risk setting.

Greenberg et al. in 2001 published the results of their study on 17 average and high-risk patients treated with radiotherapy associated to Packer’s chemotherapeutic regimen. They failed to show that chemotherapy is effective when added to craniospinal radiation in adult patients with medulloblastoma. Relapse-free survival and overall survival did not reach statistical significance. Furthermore, the patients experienced considerably greater chemotherapy-related toxicity than did children treated on an identical protocol [10]. In a study by Friedrich et al. adult patients were treated with lomustine, vincristine and cisplatin for 8 cycles after radiotherapy according to the HIT 2000 protocol. In this study the incidence of grade ≥ 3 hematological adverse events was 58% and grade ≥ 2 neurotoxicity was 69%. More than half of patients were not able to perform the planned 8 cycles and experienced dose delays and reductions [11]. In NOA-07 multicenter pilot study 25 adult patients received combined craniospinal irradiation with vincristine followed by 8 cycles maintenance chemotherapy with cisplatin, lomustine and vincristine. Seventy percent of patients tolerated 4 cycles of chemotherapy, but treatment was withdrawn or dose was reduced in almost 60% of patients after cycle 4 due to side effects. Leucopenia and thrombocytopenia were the any grade major toxicity. Polyneuropathy and ototoxicity were the only grade 3 and 4 non hematological toxicities (40% of patients). The authors concluded that this regimen was not feasible in adult patients [12]**.**

About chemotherapeutic schedules, we found that cisplatin or carboplatin plus etoposide have a favorable toxicity profile, avoiding hematologic toxicities due to the cumulative dose of nitrosoureas and are more feasible in adult than pediatric protocols [3, 13, 14].

In our previous study we showed a trend for improved OS for average risk patients treated with chemotherapy after a median follow up of 10 years (*p* = 0.079) [15]**.** In the present study with more patients and a longer follow up time we showed a statistically significant survival benefit from adding chemotherapy in terms of OS and PFS (*p* = 0.05 for PFS and 0.02 for OS). The patients treated with radiotherapy and chemotherapy had PFS-15 and OS-15 rates of 82.3 and 89.3% versus PFS-15 and OS-15 rates of 38.5 and 52.0% with radiotherapy alone.

We did not find a higher toxicity from the addition of chemotherapy compared to toxicity rates reported by in literature [11, 12]. By analyzing safety data, as expected, the main toxicities were neutropenia and thrombocytopenia and among all patients receiving chemotherapy we reported a grade ≥ 3 hematologic adverse events incidence rate of 42% compared to 16% reported in the group of patients who received radiotherapy alone and a negligible rate of grade ≥ 3 gastrointestinal effects (8% for grade 3 nausea). The events were reversible in all cases and only for 3 patients (13%) we recurred to the use of granulocytes- colony stimulating factors. None died for adverse events related to chemotherapy.

As in children, a possibility to reduce acute treatment-related toxicities also in adult patients is to decrease the dose of radiotherapy in patients receiving chemotherapy through the development of new radiation therapy technologies such as proton beam cranio-spinal irradiation. The increasing use of these new strategies in the next future could allow to obtain an increasing survival from the addition of chemotherapy to adjuvant radiotherapy with a better profile of gastrointestinal and hematologic safety [16].

## Conclusions

After a median follow up of 12.5 years,we found a statistically significant benefit from addition of adjuvant chemotherapy in the management of average-risk medulloblastoma patients. This benefit can be assessed only after many years (15 or more) from surgery since tumor relapses are often delayed in average-risk disease. Many questions remain open about timing and schedules of chemotherapy and the possibility to reduce radiotherapy doses and, consequently, toxicities. Further research is needed to eventually standardize the role of chemotherapy for this rare group of patients.

## Data Availability

The datasets used and/or analysed during the current study are available from the corresponding author on reasonable request.

## Abbreviations

PFS: Progression-free survival
OS: Overall survival
CNS: Central nervous system
MRI: Magnetic resonance imaging
CSF: Cerebro-spinal fluid
RT: Radiotherapy
CT: Chemotherapy
